# Affirmative action in healthcare resource allocation: Vaccines, ventilators and race

**DOI:** 10.1111/bioe.13067

**Published:** 2022-08-01

**Authors:** Hazem Zohny, Ben Davies, Dominic Wilkinson

**Affiliations:** ^1^ Oxford Uehiro Centre for Practical Ethics, Faculty of Philosophy University of Oxford Oxford United Kingdom of Great Britain and Northern Ireland; ^2^ John Radcliffe Hospital Oxford United Kingdom of Great Britain and Northern Ireland; ^3^ Murdoch Children's Research Institute Melbourne Australia

**Keywords:** affirmative action, COVID‐19, distributive justice, race

## Abstract

This article is about the potential justification for deploying some form of affirmative action (AA) in the context of healthcare, and in particular in relation to the pandemic. We call this Affirmative Action in healthcare Resource Allocation (AARA). Specifically, we aim to investigate whether the rationale and justifications for using prioritization policies based on race in education and employment apply in a healthcare setting, and in particular to the COVID‐19 pandemic. We concentrate in this article on vaccines and ventilators because these are both highly scarce resources in the pandemic, and there has been a need to develop policies for allocating them. However, as will become clear, the ethical considerations relating to them may diverge. We first set out two rationales for AAs and what they might entail in a healthcare setting. We then consider some disanalogies between AA and AARA, as well as the different implications of AARA for allocating ventilators as opposed to vaccines. Finally, we consider some of the practical ways in which AARA could be implemented, and conclude by responding to some key objections.

## 1 INTRODUCTION

The COVID‐19 pandemic has disproportionately affected disadvantaged racial and ethnic minorities. In the U.K., in the first phase of the pandemic, people from particular racial groups (Black individuals and people from some South Asian backgrounds) had a higher risk of being hospitalized from COVID‐19. Black men were 4.2 times more likely to die than White.^1^ Even after taking into account other risk factors, COVID‐19‐related death for males and females of Black ethnicity in the U.K. occurred at almost double the rate of those of White ethnicity.^2^ In the United States, the Centre for Disease Control reported a similar trend: Black and Hispanic people were five times more likely to be hospitalized from COVID‐19 than White people, and three times more likely to die.^3^

In response to these disparities, some argue we should allocate pandemic resources, such as ventilators and vaccines, in ways that explicitly prioritize members of disadvantaged racial groups. For instance, some legal scholars suggest that, because of racial inequality in housing, employment and access to healthcare, we are required to prioritize vaccine access by race.^4^ Influential policy advocates similarly argue that, in the United States, after healthcare workers, Black people ‘and many other people of color’ should be next in terms of vaccine distribution.^5^ Ethicists have also argued that we should consider placing people of colour high on the list for vaccine priority, and give more weight to individual members of disadvantaged racial groups so that they have a higher chance of accessing ventilators.^6^ As one physician put it, a patient from a disadvantaged racial group ‘coming in needing a ventilator may have co‐morbidities because she has already lived a life of deprivation and discrimination. It might be worth giving her *more* resources now, precisely because she has received *less* resources in the past. It is a form of affirmative action (AA) of medical resources, if you will’.^7^

What follows is an evaluation of this prospect of using AA in the context of healthcare, and specifically in relation to the COVID‐19 pandemic. Our goal is not to settle the normative questions raised by such a prospect, but to clarify the various rationales for it, and how their implications might differ from the contexts of education and employment. We also consider what such a policy might look like if deployed in practice and how it might be balanced against competing values (Section 6). Three clarifications are made before commencing. First, while we aim to consider the different normative rationales and implications of healthcare prioritization based on race, we will not directly address the legality of any such prioritization scheme, only its ethics. Second, because our aim is to describe a general phenomenon, and because which particular racial or ethnic groups are disadvantaged with respect to COVID‐19 varies by geographical region, we use the phrase ‘disadvantaged racial groups’ except when referring to specific examples. Third, for the purposes of this article we will use a broad definition of AA. We elaborate on this in Section 3, but to set the scope from the onset, we rely on the definition used by Lippert‐Rasmussen:

Affirmative action (AA): A policy that ultimately aims at reasonably increasing the representation of minorities in the relevant area or reasonably addressing the disadvantages they suffer in the relevant area.^8^

## 2 DISCRIMINATION AND DISADVANTAGE

Before delving into the prospect of AARA, it is worth clarifying what is meant by a disadvantaged racial group in the context of the pandemic. What racial groups are, and whether they even exist, is philosophically contested.^9^ Yet even those who think we ought to discard race as a biological concept can accept that people's lives are affected in significant ways by being ‘racialized’ or placed into racial categories, regardless of the specific ontology of these categories. This means that racial categories can be important in identifying patterns of health‐related disadvantage.

As already summarized, it is clear that members of various racial groups have experienced significant disadvantage during this pandemic. The normative significance of that disadvantage does not rely on the grouping itself having any objective validity: people can be affected by inappropriate categorization. On the contrary, the nature of social disadvantage arising from social interactions, choices and behaviour generates part of the ethical argument in favour of AA.

A person's race may give rise to disadvantage in the context of healthcare in several ways. It is common in both law and theory to distinguish ‘direct' and ‘indirect’ discrimination, including racial discrimination. Direct racial discrimination occurs when an individual is disadvantaged on the basis of being racialized; that is, placed into a racial category. As well as obvious examples of deliberate discrimination, direct racism includes cases involving the biases of otherwise well‐meaning individuals. For instance, the Association of American Medical Colleges has found that ‘half of white medical trainees believe such myths as black people have thicker skin or less sensitive nerve endings than white people’, which may have an impact on the treatment of Black patients.^10^ Because these misbeliefs involve disadvantaging Black people on the basis of their race, they constitute direct racism, even if it is unintentional.

Indirect discrimination covers cases where practices that are apparently neutral put one or more racial groups at a disproportionate disadvantage.^11^ Just as direct racism in access to other social goods has a significant impact on health, so too may indirect racism that leads to racialized access to high‐paying employment, education and other opportunities. Within a healthcare setting itself, arguments against the use of ‘colourblind’ allocation algorithms are one example of an appeal to the indirect impact of an apparently neutral policy.^12^

## 3 AA IN HEALTHCARE

There are two central ways in which AA, generally construed, has been justified.^13^ One is through the idea of ‘representation’; one might think that key areas of society such as education and employment ought to reflect the makeup of that society with respect to socially salient categories,^14^ and that such a distribution will have instrumentally beneficial effects, such as creating role models.^15^

This rationale does not straightforwardly translate to a healthcare setting. The equivalent of being ‘underrepresented’ in health is having worse health‐related outcomes, and as a consequence a lower chance of accessing certain goods associated with good health, including quality of life and life expectancy. In the context of the pandemic specifically, this could mean a higher chance of contracting the virus, a higher chance of more severe COVID‐19, and/or a higher chance of adverse outcomes from the infection, including dying. In that regard, we might say that members of disadvantaged racial and ethnic groups are under‐represented in those surviving the pandemic.

An alternative rationale for AA that may translate more easily is the correction of past and present injustice. In employment and education, the basic thinking is that injustice has created barriers against members of disadvantaged racial groups accessing key social goods.^16^ This correction may take two forms. First, insofar as past and present injustice means that members of oppressed racial groups have unequal opportunities, AA may aim, as far as possible, to restore equal opportunity. Less commonly, and more controversially, the approach may aim to *compensate* for past injustice over and above restoring equality of opportunity.^17^ This rationale seems relevant to healthcare, because past and present injustice might impact the health of individuals from disadvantaged racial groups in myriad ways.

We can distinguish two forms of AARA, as follows.^18^

*Weak AARA*: A policy of allocating scarce resources that seeks to provide strictly equal chances of allocation by encouraging participation of the disadvantaged racial group and seeking to reduce risk of bias in selection among those who are (roughly) equally qualified for allocation.

*Strong AARA*: A policy of preferentially allocating scarce resources to a disadvantaged racial group.

For example, with vaccines, weak AARA might pay particular attention to access arrangements (e.g., locations and opening times of vaccination clinics) as well as careful attention to the information provided about vaccines. This would help ensure equal chances of receiving a vaccine among those who best fit the existing allocation criteria. In contrast, a strong form of AARA would give preferential access to the vaccine to members of disadvantaged racial groups in a community, so that they receive it earlier than otherwise similarly placed members of other racial groups.

For ventilators, a weak form of AARA would seek to ensure that clinical assessments of patients for intensive care admission were not subject to bias. Efforts might be made to ensure that there are equivalent numbers of ventilators (and qualified medical staff) in hospitals servicing disadvantaged racial groups.

Strong AARA for ventilators would mean prioritizing patients from a disadvantaged racial group over patients with equivalent or perhaps even more serious medical factors. In healthcare (as in other areas), there is a compelling ethical argument in favour of weak AARA. It goes without saying that allocation should seek to avoid conscious or unconscious bias, and that we should ensure equal access to consideration. However, the more controversial question is about the place of strong AARA. We will therefore focus for the remainder of this article on the case for strong AARA.

## 4 TWO KEY DISANALOGIES

One argument in favour of strong AARA is an argument from analogy. There is marked inequality in access to employment/education for individuals from disadvantaged racial groups, with significant negative consequences for members of those groups. AA can be justified in employment/education as a means of addressing this inequality. There appears to be similar inequality in the health domain, also with serious negative consequences for those affected. Assuming that there is nothing special about healthcare then, if AA is justified, AARA seems similarly justified. However, to the extent that the argument for AARA is based on analogy, there may be some relevant disanalogies.

### 4.1 Criteria for selection

The first and most obvious is between the criteria for selecting candidates in education and employment, and those for allocating healthcare resources to treat patients, especially scarce resources in a pandemic. The selection criteria in education and employment generally relate to having certain qualifications or ability (such as grades, skills or expertise), and these may relate more broadly to desert. They are often necessarily competitive, such that the most qualified, or most able, are offered a position.

In contrast, the selection criteria required for using healthcare resources are unrelated to achievement or talents, but are often related to, among other things, the patient's need for the resource. In general, the scarcer a particular healthcare resource is, the greater the threshold of need will be for receiving the resource. When a healthcare resource is in short supply, patients with a low need for treatment will usually not be able to access it.

One implication of the latter principle is that healthcare allocation might be already designed to address disadvantage. Patients with worse health will usually have greater health needs. That will often lead to them having higher priority for medical treatment. If that is the case, there would be no need for a separate process of AA in allocation.

However, allocation of scarce medical resources is not based purely on need, and will not always prioritize the disadvantaged. In some circumstances, patients with worse health states or with health‐related disadvantages will benefit less from access to medical treatment compared with others. Resource allocation is also influenced by a desire to achieve the greatest total healthcare benefit from treatment. When there is a severe scarcity of medical resources, giving absolute priority to the worst off would potentially lead to much worse health outcomes overall.^19^

This suggests that the analogy of who is best ‘qualified’ for accessing medical treatment may require some combination of need *and* capacity to benefit. Thus, although the criteria for candidate selection might superficially seem to avoid the need for AA in healthcare—because it is already focused on alleviating disadvantage—the fact that meeting health needs effectively only imperfectly tracks disadvantage means that this apparent disanalogy has less force.

On the other hand, the rationale for including effectiveness as a criterion for allocation suggests that giving too strong a priority to the most disadvantaged may have an overall undesirable effect on our ability to treat others, *including* other members of disadvantaged groups. This would mean that AARA should not give absolute priority based on need, though this does not itself differentiate it from existing forms of AA.

### 4.2 Consequences

Another potential disanalogy is the impact of AA and AARA. It is difficult to assess exactly how AA policies in education and employment have affected those from comparatively advantaged groups. By and large, the evidence suggests that White men did not miss out on attending higher education or on finding jobs—they merely went elsewhere.^20^ But even if it were the case that some individuals from advantaged groups miss out on opportunities as a consequence of AA, the nature of such choices tends to be either zero‐ or positive‐sum: a loss for one individual is counterbalanced by an equivalent gain for another; or, the loss is outweighed by an even greater gain for another (to the extent that education increases further gains in other domains of life, such as health).^21^

In contrast, the consequence of allocation in the setting of a pandemic has wider implications. Where resources are critically limited, a policy of allocating ventilators to patients from a disadvantaged background who have a lower chance of survival would likely lead to greater numbers of deaths overall. Moreover, when it comes to ventilator allocation, there is a further complication. Patients who have a greater pre‐existing health disadvantage may be at higher risk of dying, but they may also be at higher risk of prolonged treatment in intensive care. The latter is important in a situation where intensive care access is critically limited, because allocation of treatment to one patient may come at the cost of several patients being unable to access intensive care. It could therefore come at the cost of a further reduction in overall survival from COVID‐19, including among those from disadvantaged racial groups.^22^ This does not by itself mean that AARA lacks justification, but it is a cost that must be taken into account.

## 5 DIFFERENTIAL IMPLICATIONS—VACCINES VERSUS VENTILATORS

Whether AARA is overall ethically justified will depend on how we weigh up some of these concerns against the positive reasons. Of relevance, some of the above considerations apply in a different way, or to a different degree, to vaccines compared with ventilators.

### 5.1 Ventilators

We noted that pursuing strong AARA in the setting of a severe shortage of ventilators will likely reduce the total amount of healthcare benefit that those ventilators can do, and may result in more patients dying overall. This means that in the context of ventilators, there are two central values that are in tension: increasing overall health benefit, and justice. We assume that neither value can be prioritized fully over the other. It would be wrong to maximize efficacy by entirely ignoring harder‐to‐benefit members of disadvantaged groups; but it would also be wrong to exclusively prioritize extremely disadvantaged patients who would likely die even with access to ventilators.

### 5.2 Vaccines

However, these concerns do not apply, or do not apply as strongly, to vaccination programmes.

This is in part because not receiving a vaccine, or receiving it later, does not entail the same kind of cost to the individual. Those deprioritized for a ventilator will typically not be able to wait for later treatment, and will suffer serious health consequences or die as a result of not receiving timely respiratory support. Many deprioritized individuals can continue to isolate or shield while waiting to receive the vaccine later, though the ease of this depends on people's personal and economic circumstances. Arguably, part of the reason for the higher incidence of COVID‐19 deaths among people from some disadvantaged racial groups is that shielding is not always an option for the economically vulnerable. This potentially means that those from more advantaged groups are in a better position to withstand the costs of waiting for vaccination.

Ultimately, with vaccination the ethical values in allocation are not necessarily in conflict. On the contrary, the more vulnerable or in need of a vaccine an individual is, the more a vaccine could benefit them. If disadvantaged racial minorities are generally more vulnerable to coronavirus, they already have a correspondingly greater claim to being prioritized when it comes to vaccine distribution. Moreover, such prioritization would not obviously result in worse healthcare outcomes. On the contrary, it would be directing the vaccine to those most at risk from the virus, and (potentially at least) would therefore reduce overall cases of severe illness and death.

## 6 BALANCING ETHICAL VALUES—IMPLICATIONS FOR AARA

One key reason why allocation of resources is ethically fraught is that it frequently requires a trade‐off between competing ethical values, particularly those of fairness and benefit.^23^ The difficulty is finding the right balance between these. Different individuals and societies will reach different conclusions about this. That is partly because of factual circumstances (e.g., different degrees of inequality in health outcomes associated with race), but it will also reflect different ethical weighting of the relevant values.

The question of prioritizing members of a disadvantaged racial group in resource allocation represents one instance of the wider ethical problem, and as such it may be possible to apply some lessons drawn from other areas. A starting point is to identify areas of ethical convergence—where there is no conflict between values. We have suggested above that AARA in vaccine allocation (prioritizing those from racial groups at higher risk of contracting the virus and/or of severe consequences if they do) is clearly ethically justified for just this reason. For healthcare systems that have a list of vaccine priority groups (Table 1), this might be achieved through the concept of ‘risk equivalence’.

**Table 1:** Vaccine priority

**Vaccine priority groups and risk equivalence** In the U.K., for instance, COVID‐19 vaccination is being rolled out on the basis of risk groups.^a^
1. Residents in a care home for older adults and their carers 2. All those 80 years of age and over and in frontline health and social care workers 3. All those 75 years of age and over 4. All those 70 years of age and over, and clinically extremely vulnerable individuals 5. All those 65 years of age and over 6. All individuals aged 16 years to 64 years with underlying health conditions that put them at higher risk of serious disease and mortality 7. All those 60 years of age and over 8. All those 55 years of age and over 9. All those 50 years of age and over 10. Rest of the population (to be determined) The idea of risk equivalence is that patients with a similar risk of serious illness from the virus should receive a similar priority for access to the vaccine. If people from a disadvantaged racial group have increased risk because of their race—or whatever race acts as a proxy for—they should be lifted up one or more categories. For example, if people from a racial minority group who are aged 55–60 have the same risk of serious illness as people aged 60–65 from the comparably less‐disadvantaged racial majority, it would be ethical for them to receive equivalent priority to the older group.^b^

^a^ COVID‐19 vaccination first phase priority groups. *Public Health England*. https://www.gov.uk/government/publications/covid-19-vaccination-care-home-and-healthcare-settings-posters/covid-19-vaccination-first-phase-priority-groups [Access 25/2/2021]

^b^ Of note, there are more complicated potential cases relating to vaccination where the ethical values would not converge. For example, if a vaccine were less effective for members of a disadvantaged group who were at higher risk from a virus, then prioritization of this group might come at the cost of less benefit overall from the vaccine. We will not discuss these more complicated cases in any detail, but the principles outlined in the next section would potentially apply.

Next, one way of incorporating ethical values (such as priority for the disadvantaged) is to look for cases where other factors are evenly balanced. In a ‘tie‐break’ situation, where two candidates for a position are evenly matched in their suitability for employment but one of them is a member of a disadvantaged group, it seems most clear that AA might justifiably lead to the minority applicant receiving the position. By analogy, if there were a single available ventilator, and a health professional needed to choose between patients who appeared otherwise to have an identical prognosis, it may be justifiable to take into account the fact that one of these patients came from a disadvantaged racial group.^24^ That decision would give some weight to the ethical value of justice.

Of course, in reality, decisions about the allocation of ventilators rarely come down to choices between two patients for a single ventilator. Instead, health professionals may need to decide whether an individual patient presenting with respiratory failure falls above or below a requisite ‘threshold’ of benefit for admission. Such decisions are complex and potentially subjective. For that reason, they are potentially vulnerable to bias, and (as mentioned above) there is a clear ethical case for seeking to avoid that.^25^ However, as an extension of the ‘tie‐break’ suggestion, it may also be justifiable in borderline cases (where a patient's prognosis appears close to the threshold for admission to intensive care) to give some additional weight to the fact that they belong to a disadvantaged group (see Table 2).

**Table 2:** Triage scoring

Triage scoring
One potential way of applying AARA might be in a situation where a healthcare system used a numerical score to help with decisions about allocation of resources. Such scoring systems are sometimes used in decisions about organ allocation, and such a score was contemplated (though ultimately not implemented) by the U.K. National Health Service (NHS) in the early stages of the COVID‐19 pandemic.^a^ The proposed NHS Decision Support score combined elements of age, clinical frailty and comorbidities into a score out of 28. The idea was that patients who were assessed as having a score of more than 8 would not be treated with invasive ventilation/intensive care.
Giving some weight to justice and racial disadvantage might mean that decision‐support scores should be adjusted. For example, those from a particular racial group might have a point subtracted from their triage score (so that they are more likely to be eligible for intensive care).

^a^ Foster, P., Staton B., & Rovnick, N. (2020, April 13). NHS ‘score’ tool to decide which patients receive critical care. *Financial Times*. https://www.ft.com/content/d738b2c6-000a-421b-9dbd-f85e6b333684 [Access 19/7/2021]

Exactly how this form of strong AARA for ventilator allocation would be applied will depend on the circumstances of a particular society. If a society has ample availability of ventilators (because they have a well‐resourced healthcare system and/or they have a relatively low number of cases of patients with severe COVID‐19), few patients would be denied admission to intensive care. There would therefore be few cases where there would be a need to consider prior racial disadvantage in allocation. At the other extreme, in a low‐resource country with very few available ventilators and a severe surge in demand for intensive care, it may be that there is little opportunity to take into account prior disadvantage.

However, balancing ethical values entails the potential for cases where the fact that an individual patient is from a disadvantaged racial group will not lead to allocation of a ventilator. This might apply where a patient's outlook is significantly below the ordinary threshold for admission to intensive care. There is an even clearer conflict between ethical values in situations where access for one patient (from a disadvantaged racial group) would potentially come at the cost of several other patients. That applies in relation to the duration of support in intensive care. This suggests that in ‘different number cases’—where there is a need to balance the needs of several patients against a single patient—ethical parity (and balancing different ethical values) would limit the role of strong AARA and prioritize the greater number of patients—in this case, those needing a shorter duration of treatment.^26^

## 7 OBJECTIONS TO AARA

### 7.1 Race as a morally irrelevant characteristic

One potential objection is that race is not relevant; rather, race correlates with a number of other factors (e.g., poverty, worse overall health) that are morally relevant. Thus, one might insist, we should prioritize based on those factors, not on race.

However, this ignores several important points. First, members of disadvantaged racial groups may be affected by clusters of these other factors, meaning that groups such as the economically badly off are also racially structured in terms of vulnerability. Second, the instruction to focus only on the factors that directly affect vulnerability assumes that we know what those factors are, and precisely how vulnerable each individual in these categories is. Because membership of certain racial groups correlates with vulnerability to COVID‐19, it is no less plausible a prioritization category. Finally, some will argue that because greater vulnerability of members of racially disadvantaged groups is due to historic and present injustice, there are additional reasons for prioritization (though this may also apply to other groups, such as those who are economically disadvantaged).

### 7.2 Stereotyping

While there is ample evidence that, overall, members of particular racial groups have been disadvantaged during the COVID‐19 pandemic, that disadvantage will not have been experienced equally. Moreover, there is a spectrum of health advantage, and there is likely to be some overlap. Indeed, some members of a disadvantaged group may actually be better off than some members of an advantaged racial ethnic group. A policy of strong AA would potentially worsen social injustice for some.

This sort of concern clearly applies to AA in education and employment. In that setting, those who defend AA advocates typically reply that while AA is imperfect, it may still be permissible because of the importance of AA's social goals. Likewise, in the setting of healthcare allocation, while a rule giving higher vaccine priority to members of a racial group would advantage some who do not need priority (and disadvantage others who do), overall such a rule would serve its goal of prioritizing those at highest risk. What is more, in an allocation system that includes multiple factors, other considerations will help to reduce uneven disadvantage. For example, in the vaccine priority list described in Table 1, the consideration of underlying health conditions would lead to higher priority.

### 7.3 Perverse effects

One potential worry about AA is that it may lead individuals from disadvantaged racial groups, or their co‐workers or fellow students, to feel that they have not ‘earned’ their place.^27^ Even if this is not a justified reaction, it is still a cost to bear in mind: a well‐intentioned policy might make things worse in some ways for its intended beneficiaries, or might be misperceived.

A potentially analogous effect could occur in vaccination. A very strong form of AARA that gave members of disadvantaged racial groups absolute priority for vaccine access might exacerbate existing issues of mistrust. For instance, it might be thought that racial minorities are being used to ‘test’ an unproven vaccine so it can be safely given to White patients. This is not an absolute objection to AARA, but rather a reminder that any such policy must be effectively managed and communicated and will need to involve discussion and input from members of the relevant groups.

### 7.4 Epistemic challenges in prioritization

One challenge for AARA is that the boundary between advantaged and disadvantaged racial groups is not clearly defined. For example, if a vaccine or a ventilator allocation process were to give higher priority to Black patients, how should race be determined? It might be straightforward in some cases, but what of those who have a multi‐racial background or who belong to smaller minority racial groups?

One approach in relation to employment or education is for individuals to identify which racial group they identify with. However, self‐identification might not be possible in the context of seriously ill patients needing a ventilator. Moreover, in some cases self‐identification of race is disputed,^28^ or challenged by those both in disadvantaged and in non‐disadvantaged groups.

Because this problem affects AA in all areas, and is not unique to AARA, we do not intend to resolve it here, except to note that if this problem does not preclude AA in other areas, it should not necessarily preclude AARA. However, one point to note is that the other factors typically (and uncontroversially) included in healthcare allocation are also often vague, and include borderline cases that are hard to classify.

### 7.5 Other types of disadvantage

Finally, if AARA were to be implemented, one question is whether or why it should be applied to race alone, and not to other factors associated with healthcare disadvantage. For example, obesity and male sex are associated with higher risk of severe COVID‐19 and higher mortality rates.^29^ Should these factors also be taken into account in vaccine or ventilator allocation? Should men or obese individuals have access to the vaccine sooner than women or those of healthy weight?

One response would be to include in allocation any factors that are relevant either to the need for treatment or to the benefit of treatment. That would mean (relatively uncontroversially) that factors that increase the risk of severe COVID‐19 might be taken into account in giving priority for vaccination. As noted above, allocation for ventilators is more ethically complicated, because the higher burden of illness might simultaneously give reasons for higher and lower priority.

A different response would be to suggest that there is something different about the health disadvantage associated with race (compared, for example, to that associated with male sex). Whereas the worse health outcome from COVID‐19 for members of disadvantaged racial groups appears to coincide with social injustice, the opposite is the case for men overall as a social group (because they have in general been the beneficiaries of gendered social injustice). On this view, AARA would be justified in selectively applying to groups that have been the victims of social injustice.

Again, our aim here is not to resolve this question, and it is a problem that affects other forms of AA. The decision about which factors to include in AARA will necessarily be specific to a particular community—it will reflect the history of both discrimination and injustice (and which groups have been particularly disadvantaged) and will reflect the ethical priorities and values of the wider community.

## 8 CONCLUSION

AA has always been controversial and is likely to be particularly controversial in a fast‐moving and far from fully understood global health (and, in turn, social and economic) crisis. This article has not aimed to settle the normative questions raised by AARA, let alone its pragmatic or legal constraints, but to clarify what such policies might look like, and to what extent they differ from the rationales for AA in employment and education.

One key conclusion is that, while healthcare allocation is already designed to prioritize patients with worse health, this does not necessarily eliminate the case for AA in healthcare. Another is that AARA may apply differently for different healthcare resources. The ethical values in allocating scarce vaccines do not conflict in the same way as they do with ventilators. For the latter, strong prioritization of patients with a worse prognosis would entail reduction in overall survival from COVID‐19, including among those from disadvantaged racial groups.

In terms of practical application, we suggested that by appealing to risk equivalence between groups, AARA may have a role to play in vaccine distribution, as well as potentially serving as a tiebreaker in the allocation of limited resources such as ventilators. However, how exactly these forms of AARA will be applied will vary between societies in reflection of the availability of resources such as vaccines and ventilators, the degree of historic and ongoing discrimination or injustice to which particular racial groups have been subjected, and the way that societies choose to balance conflicting ethical values.

## CONFLICT OF INTEREST STATEMENT

This study was commissioned and paid for by the World Health Organization (WHO). Copyright of the original work on which this article is based belongs to WHO. The authors have been given permission to publish this article.

The authors alone are responsible for the views expressed in this publication and they do not necessarily represent the views, decisions or policies of the WHO.

Dominic Wilkinson was supported for this work by a grant from the Wellcome Trust, 203132/Z/16/Z, and by the UK Research and Innovation/Arts and Humanities Resaerch Counci‐funded U.K. Ethics Accelerator project, grant number AH/V013947/1. The funders had no role in the preparation of this manuscript or in the decision to submit for publication

## Footnotes

1 Public Health England (2020). *Disparities in the risk and outcomes of COVID‐19*. Retrieved February 26, 2021, from https://assets.publishing.service.gov.uk/government/uploads/system/uploads/attachment_data/file/908434/Disparities_in_the_risk_and_outcomes_of_COVID_August_2020_update.pdf

2 Ibid.

3 Center for Disease Control and Prevention. (2020). *COVID‐19 in racial and ethnic minority groups*. Centers for Disease Control and Prevention.

4 Twohey, M. (2020). Who gets a vaccine first? U.S. considers race in coronavirus plans. *New York Times, July 9, 2020*. Retrieved February 26, 2021, from https://www.nytimes.com/2020/07/09/us/coronavirus-vaccine.html

5 Jagannathan, M. (2020). *Should Black and Latino people get priority access to a COVID‐19 vaccine?* Marketwatch. Retrieved February 25, 2021, from https://www.marketwatch.com/story/should-black-and-latino-people-get-priority-access-to-a-covid-19-vaccine-2020-07-16

6 Sederstrom, N. (2020). *The ‘Give Back’: Is there room for it?* Bioethics.net. Retrieved February 23, 2021, from http://www.bioethics.net/2020/07/the-give-back-is-there-room-for-it/

7 McLane, H. (2020). *A disturbing medical consensus is growing: Here's what it could mean for Black patients with coronavirus*. Whyy.org. Retrieved February 20, 2021, from https://whyy.org/articles/a-disturbing-medical-consensus-is-growing-heres-what-it-could-mean-for-black-patients-with-coronavirus/

8 Lippert‐Rasmussen, K. (2020). *Making sense of affirmative action* (p. 12). Oxford University Press. Note: We acknowledge that some definitions of affirmative action are more restrictive than this. The policies that we describe in relation to vaccine/ventilator allocation would not fall within definitions of AA that restrict the domain to education, employment or culture, or that specifically restrict it to policies that aim to compensate for past exclusion and injustice.

9 Glasgow, J., Haslanger, S., Jeffers, C., & Spencer, Q. (2019). *What is race? Four philosophical views*. Oxford University Press.

10 Hofman, K. M., Trawalter, S., Axt, J. R., & Oliver, M. N. (2016). Racial bias in pain assessment and treatment recommendations, and false beliefs about biological differences between Blacks and Whites. *Proceedings of the National Academy of Sciences of the United States of America*, *113*(16), 4296–4301.

11 Khaitan, T. (2017). Indirect discrimination. In K. Lippert‐Rasmussen (Ed.), *The Routledge handbook of the ethics of discrimination* (pp. 30–41). Routledge.

12 For an example of this relevant to the COVID‐19 pandemic, see Williams, J. C., Anderson, N., Mathis, M., Sanford, E., Eugene, J., & Isom, J. (2020). Colorblind algorithms: Racism in the era of COVID‐19. *Journal of the National Medical Association*, *112*(5), 550–552.

13 Allen, A. L. (2011). Was I entitled or should I apologize? Affirmative action going forward. *The Journal of Ethics*, *15*(3), 253–263.

14 For instance, see Section 8 in Fullinwider, R. (2018). Affirmative action. In E. N. Zalta (Ed.), *The Stanford encyclopedia of philosophy*. Stanford University. Retrievd from https://plato.stanford.edu/entries/affirmative-action/

15 Boxill, B. (2009). Discrimination, affirmative action, and diversity in business. In G. G. Brenkert (Ed.), *The Oxford handbook of business ethics* (pp. 535–562). Oxford University Press.

16 Jones, G. (1985). Preferential treatment and the allocation of scarce medical resources. *The Philosophical Quarterly*, *35*(141), 382–393; Thomson, J. J. (1973). Preferential hiring. *Philosophy & Public Affairs*, *2*(4), 364–384; Goldman, A. H. (1976). Affirmative action. *Philosophy & Public Affairs*, *5*(2), 178–195; Appiah, K. A. (2011). ‘Group Rights’ and racial affirmative action. *The Journal of Ethics*, *15*(3), 265–280.

17 Overall, C. (2005). *Aging, death, and human longevity: A philosophical inquiry* (pp. 200–205). University of California Press; Boxill, B. (2009). Discrimination, affirmative action, and diversity in business. In G. Brenkert (Ed.), *The Oxford handbook of business ethics (pp. 535–562)*. Oxford University Press.

18 This distinction adapts a more general distinction about affirmative action made in: Nagel, T. (1981, Jun 18). *A defense of affirmative action*. *Testimony before the Subcommittee on the Constitution of the Senate Judiciary Committee*. Retrieved from https://pdfs.semanticscholar.org/45c4/b8ee0de4a673ec82dbb7cadfd503ac77d150.pdf

19 Purely need‐based allocation leads to the ‘bottomless pit’ objection in healthcare distribution. See Juth, N. (2015). Challenges for principles of need in health care. *Health Care Analysis*, *23*(1), 73–87.

20 Urofsky, M. I. (2020). *The affirmative action puzzle: A living history from reconstruction to today* (Illustrated ed.). Alfred A. Knopf.

21 We thank an anonymous reviewer for highlighting the possibility of positive‐sum outcomes with AA in education.

22 Because patients from disadvantaged groups may be at higher risk of needing treatment, they would potentially be disproportionately affected if intensive care unit bed numbers were reduced (because of allocating treatment to patients who end up with a significantly longer length of stay).

23 Wilkinson, D. J., & Savulescu, J. (2020). Prioritization and parity. In A. Cureton & D. Wasserman (Eds.), *The Oxford handbook of philosophy and disability (pp. 669–692)*. Oxford University Press.

24 In this hypothetical situation, the two patients need not have an identical outlook—rather that the other relevant considerations are ‘on a par’. For further discussion of the notion of parity in allocation decisions, see Wilkinson & Savulescu (pp. 669–692), op. cit. note 23.

25 Indeed, the COVID‐19 pandemic has instigated a significant literature assessing the various triage protocols and their underlying rationales and criteria and proposing new ones. See for instance Jöbges, S., Vinay, R., Luyckx, V. A., & Biller‐Andorno, N. (2020). Recommendations on COVID‐19 triage: International comparison and ethical analysis. *Bioethics*, *34*(9), 948–959; Tolchin, B., Hull, S. C., & Kraschel, K. (2021). Triage and justice in an unjust pandemic: Ethical allocation of scarce medical resources in the setting of racial and socioeconomic disparities. *Journal of Medical Ethics*, *47*(3), 200–202; Sönmez, T., Pathak, P. A., Ünver, M. U., Persad, G., Truog, R. D., & White, D. B. (2021). Categorized priority systems: A new tool for fairly allocating scarce medical resources in the face of profound social inequities. *Chest*, *159*(3), 1294–1299.

26 Wilkinson & Savulescu, op cit. note 23.

^27^
Though see Sterba, J. (2017). Affirmative action for the future. In N. Zack (Ed.) *The Oxford handbook of philosophy and race* (pp. 537–547). Oxford University Press.

^28^
See Yang, T. (2006). Choice and fraud in racial identification: The dilemma of policing race in affirmative action, the census, and a color‐blind society. *Michigan Journal of Race and Law, 11*, 367–417.

^29^
Popkin, B., Du, S., Green, W., Beck, M. A., Algaith, T., Herbst, C. H., … Shekar, M. (2020). Individuals with obesity and COVID‐19: A global perspective on the epidemiology and biological relationships. *Obesity Reviews*, *21*(11), e13128; Peckham, H., de Gruijter, N. M., Raine, C., Radziszewska, A., Ciurtin, C., Wedderburn, L. R., … Deakin, C. T. (2020). Male sex identified by global COVID‐19 meta‐analysis as a risk factor for death and ITU admission. *Nature Communications*, *11*(1). https://doi.org/10.1038/s41467-020-19741-6

